# Contribution of Macrophage Efferocytosis to Liver Homeostasis and Disease

**DOI:** 10.3389/fimmu.2019.02670

**Published:** 2019-11-14

**Authors:** Andrea Kristina Horst, Gisa Tiegs, Linda Diehl

**Affiliations:** Institute for Experimental Immunology and Hepatology, University Medical Center Hamburg-Eppendorf, Hamburg, Germany

**Keywords:** liver injury, macrophage, efferocytosis, inflammation, resolution, apoptosis, phagocytosis, cytokines

## Abstract

The clearance of apoptotic cells is pivotal for both maintaining tissue homeostasis and returning to homeostasis after tissue injury as part of the regenerative resolution response. The liver is known for its capacity to remove aged and damaged cells from the circulation and can serve as a graveyard for effector T cells. In particular Kupffer cells are active phagocytic cells, but during hepatic inflammatory responses incoming neutrophils and monocytes may contribute to pro-inflammatory damage. To stimulate resolution of such inflammation, myeloid cell function can change, via sensing of environmental changes in the inflammatory milieu. Also, the removal of apoptotic cells via efferocytosis and the signaling pathways that are activated in macrophages/phagocytes upon their engulfment of apoptotic cells are important for a return to tissue homeostasis. Here, we will discuss, how efferocytosis mechanisms in hepatic macrophages/phagocytes may regulate tissue homeostasis and be involved in tissue regeneration in liver disease.

## Introduction

Under homeostatic conditions, new cells are produced and old cells die constantly within in the body. These aged and damaged cells need to be efficiently removed from the surroundings without inducing inflammation or tissue damage, which is achieved by a process called *efferocytosis*. Here, dying/dead cells are specifically recognized by phagocytes and subsequently engulfed. Inefficient removal of apoptotic cells is associated with the development of (auto) immune-mediated diseases (1–3) highlighting the importance of efferocytosis for homeostasis within the immune system. The liver has long been recognized as a major system for removal of aging/dying/activated cells. Under homeostatic conditions, aging erythrocytes and neutrophils, but also effector CD8^+^ T cells can be eliminated in the liver (4–7). This is mostly attributed to the activity of the organ-resident macrophages in the liver, the Kupffer cells. However, also during liver disease, both in experimental models as well as in patients, large amounts of dying cells are generated directly due to liver injury and/or following inflammatory responses. Various forms of insults leading to liver damage, such as infections, toxins or inflammation, often leads to rapid death of intrahepatic cell populations (mostly hepatocytes), making the efficient removal of such damaged cells extremely important to prevent unwarranted tissue damage and excessive inflammatory responses and support the restitution of tissue integrity. As the liver is known for its extreme and rapid capacity for regeneration upon liver injury, such as after the experimental procedure of partial hepatectomy, it must harbor efficient mechanisms and pathways to deal with large amounts of dying cells. Although cell death may occur through different mechanisms, such as apoptosis, necrosis or necroptosis (8), in this review we will focus on the role of mechanisms related to the efferocytic clearance of apoptotic cells in the context of liver immune tolerance and liver disease. Most hepatic cell types, including hepatocytes, hepatic stellate cells and liver sinusoidal endothelial cells have been reported to be able to take up apoptotic cells in some way (9, 10). However, the most important hepatic efferocytes are thought to be Kupffer cells together with other myeloid phagocytic cells, like neutrophils and monocytes, which are attracted into the liver after injury for the removal of apoptotic cells. Here, we will discuss the mechanisms and pathways involved in the process of efferocytosis in general, the role for these pathways in hepatic myeloid cells (tissue resident and migrating) and their relevance for both the induction and resolution of liver disease.

## Finding, Sensing, and Clearing Apoptotic Cells

The process of efferocytosis is essential for the clearance of apoptotic cells dying from physiological and pathological causes without arousing inflammation. Soluble “find me” signals are released during the onset of apoptosis to attract the phagocytically active myeloid subsets. The most well-known “find me” signals are lyso-phosphatidyl choline (LPC), sphingosine-1-phosphate (S1P), fractalkine (CX_3_CL1), and the nucleotides ATP and UTP. LPC is generated via the caspase-3-dependent activation of the inflammatory phospholipase A2 (iPLA2), which hydrolyses phosphatidyl choline (PC) from the cell membrane (11, 12). LPC then functions as an attractant for macrophages via the ligation of the G-protein coupled receptor G2A (13). Additionally, during apoptosis, the activity of sphingosine kinase leads to the caspase-3 dependent generation of S1P from sphingosine. Binding of S1P to sphingosine 1 phosphate receptors (S1PR1-5), expressed on macrophages and other phagocytes, leads to their attraction toward the apoptotic cells (11). Furthermore, membrane-bound or soluble CX_3_CL1 from either micro-vesicles of apoptotic cells (12), or cleaved by caspase-3 activity, acts as an attractant for restorative CX3CR1^+^ phagocytes. Finally, the release of the nucleotides ATP and UTP that function as extracellular alarmins, attract phagocytes via signaling through purinergic P2Y receptors and secretion via pannexin-1 channels.

The main recognition of apoptotic cells by phagocytes occurs via caspase-dependent expulsion of phosphatidyl serine (PS) onto the outer leaflet of the plasma membrane. This characteristic “eat-me” signal can be recognized directly via PS binding receptors on macrophages or indirectly by “bridging molecules” that bind to PS on the one hand and to their cognate receptors on the other hand to trigger engulfment of apoptotic cells. Most prominent are the bridging molecules milk fat globule epidermal growth factor VIII (MFG-E8; also lactadherin), and the vitamin K-dependent proteins growth arrest specific 6 (Gas6) and protein S (11, 14). Macrophages and dendritic cells can secrete MFG-E8, which then interacts with PS and can be recognized via the integrins αvβ3 or αvβ5. Both Gas6 and Protein S act as bridging molecules for the TAM receptor family, consisting of Tyro3, Axl, and MerTK (15). These interactions induce efficient clearance of apoptotic cells. Similarly, the binding of the complement factor C1q as an opsonin to PS serves as a bridging molecule for recognition by the scavenger receptor class F member 1 (SCARF1) (16) on phagocytes and endothelial cells, but may also as part of a protein complex containing calreticulin be involved in the initiation of efferocytosis (17). Recently, soluble CD93 was identified to act as an efferocytic opsonin by bridging PS on apoptotic cells with the complement receptor integrin αxβ2 (CD11c/CD18) (18). Experimentally, the detection of efferocytosis by phagocytes is most often probed with fluorescently labeled apoptotic cells, like dexamethasone-treated thymocytes or neutrophils that die via apoptosis upon *in vitro* culture. Ingestion of such apoptotic cells by phagocytes is then analyzed by microscopy or flow cytometry.

PS receptors are not ubiquitously expressed but are rather tissue and/or cell type-specific: For instance, the T cell immunoglobulin and mucin-domain-containing molecule (Tim) family of receptors act as PS receptors. In particular, Tim3 and Tim4 are expressed on phagocytes, such as macrophages and dendritic cells (DC) (11). Although Tim4 can bind directly to PS, actual clearance of apoptotic cells via phagocytosis requires its cooperation with other receptors, such as MerTK and/or integrin β1 (19, 20). The receptors stabilin-1 and-2 both recognize PS on apoptotic cells for engulfment and are expressed by macrophages, but are most prominently known for their role in the capture and elimination of PS-exposed damaged and/or aged erythrocytes by LSEC (9). Besides the uptake of aged erythrocytes by the asialo-glycoprotein receptors (ASGPR) by hepatocytes, this is the only liver cell-specific mechanism for efferocytosis described so far (10).

Like Tim4, stabilin-2 also requires cofactors/receptors [engulfment adapter phospho-tyrosine binding domain-containing protein 1 (GULP1) and thymosin4] for the initiation of engulfment (12). Additionally, some members of the CD300 family of type I transmembrane proteins (CD300a, CD300f, and CD300b) are capable of recognizing phosphatidyl-serine (PS) and -ethanolamine (PE), which are both exposed on the outer leaflet of the plasma membrane early during apoptosis (21–24). Knock-down or knock-out of CD300b and CD300f, respectively, results in impaired efferocytosis by macrophages (23, 24). Also, binding of the receptor for advanced glycation end products (RAGE) to PS increases the potential of macrophages to take up apoptotic cells (25). Contrary, secretion of the pro-inflammatory high mobility group box 1 (HMGB1) during inflammation interferes with RAGE-mediated efferocytosis by binding to PS (26). Similarly, secreted soluble RAGE itself can also inhibit efferocytosis by binding to and masking exposed PS for recognition by other PS receptors. Additional receptors, such as the scavenger receptors SR-A1 and SR-B1 and CD36 (27–29), are also implicated to play a role in (oxidized) PS recognition during efferocytosis, but their definite role has not been determined so far.

Not only do apoptotic cells signal phagocytes in various ways to enhance efferocytosis, healthy viable non-apoptotic cells express surface molecules that prevent efferocytosis. The transmembrane CD47 molecule constitutes such a “don't eat-me” signal via interaction with the ITIM-containing receptor Signal regulatory protein 1 alpha (SIRP1α; CD172a). This leads to the inhibition of actin cytoskeleton rearrangements necessary for phagocytosis (30). Recently, also the sialoglycoprotein CD24 (heat stable antigen) was shown to inhibit phagocytosis via interacting with and signaling via Sialic acid-binding Ig like lectin 10 (Siglec-10) on macrophages (31). In addition to the regulation of apoptotic cell clearance via “find-me,” “eat-me,” and “don't eat-me” signals for the phagocytotic activity of macrophages cross talk between these signaling pathways can modulate the efferocytosis process. For instance, signaling via the “find-me” receptors S1PR, CX_3_CR1 and “eat-me” receptor SCARF1 can enhance the release of the bridging molecules Gas6 and/or MFG-E8, but also upregulate their receptors, e.g., MerTK (11). This feed forward loop further enhances the capability of phagocytes after the engulfment of apoptotic cells has already been initiated.

## Modulation of Phagocyte Function Due to Efferocytosis

The process of efferocytosis and the subsequent signaling events are critical for the upkeep of homeostasis and even more important for the return to tissue homeostasis after tissue damage due to inflammation and/or disease [reviewed in (32) and (33)]. Cellular signaling and metabolic adaptation initiated by efferocytosis enables the return to tissue homeostasis by anti-inflammatory reprogramming of the formerly pro-inflammatory leukocytes: Signaling via the CX_3_CR1 receptor, recognizing CX_3_CL1 released from apoptotic cells, induces pro-survival signals and expression/generation of antioxidant factors. Signaling via S1PR invokes an anti-inflammatory gene expression program, including reduction of pro-inflammatory Interleukin (IL)-12 and tumor necrosis factor alpha (TNFα) whilst promoting production of anti-inflammatory mediators such as IL-10, vascular endothelial growth factor (VEGF) and prostaglandin E2 (PGE_2_) (32). Indeed, after efferocytosis, macrophages can produce several anti-inflammatory and pro-resolving lipid mediators that promote macrophage conversion toward phenotypes associated with resolution of inflammation (34). Also, the ATP released from apoptotic cells can be converted to adenosine by sequentially acting nucleotidases, and subsequent signaling through adenosine receptors suppresses production of proinflammatory mediators and chemokines (35).

In addition to the modulation of cellular signaling, efferocytosis leads to the accumulation of high amounts of cellular material in phagocytes that needs to be processed and digested. The metabolic adaptation to this high ingestion of cargo leads to the activation of several lipid sensing nuclear receptors, such as liver X receptor (LXR) and members of the peroxisome proliferator-activated receptor (PPAR) family (33, 36). The activation of these pathways in macrophages promotes anti-inflammatory reprogramming and can reinforce their efferocytic capacity by upregulation of phagocytic receptors, like MerTK, and inducing the synthesis of precursors for pro-resolving lipid mediators (33, 37). The uptake of apoptotic material is also closely linked to a non-canonical rubicon-dependent autophagy pathway, called light chain (LC)3-associated phagocytosis (LAP) (38, 39). Specific signaling via the TAM receptor Axl induces autophagy, which is not only involved in the physical clearance of apoptotic material, but also prevents NOD, LRR-, and pyrin-domain containing 3 (NLRP3) inflammasome activation and concomitantly reduces IL-1β and IL-18 production (40). Similarly, Tim4-mediated apoptotic cell engulfment connects to LAP after recognition of PS on apoptotic cells (41) and incomplete cargo digestion via defective LAP is associated with development of autoinflammation (42).

Classical activation or polarization of macrophages into M1 (pro-inflammatory, mostly induced by LPS/IFNγ) vs. M2 (alternative or anti-inflammatory, induced by the cytokines IL-4/IL-13) shows that also the cytokine/chemokine microenvironment in which phagocytes encounter apoptotic cells can modulate their cognate response. Classically, Th2 cells are major sources for the cytokines IL-4 and IL-13, and the simultaneous recognition of apoptotic cells (neutrophils) and IL-4/IL-13 stimulation of macrophages is necessary for the induction of an anti-inflammatory, pro-resolving tissue repair gene signature (43). Congruently, expulsion of the helminth *N. Brasiliensis* was unsuccessful in mice lacking the TAM receptors Axl and MerTK even though the capacity to produce IL-4 and IL-13 was not affected (43). IL-13 production is also essential for the induction of efferocytosis in macrophages by regulatory T cells (Treg) during resolution of a zymosan-induced model of peritonitis (44). Treg-derived IL-13 promotes autocrine IL-10 induction in macrophages leading to Vav1-Rac1-dependent phagosome formation thus promoting their capacity to phagocytose apoptotic cells (44). Although anti-inflammatory mediators, like IL-13 and IL-10, can augment efferocytosis in macrophages, not all pro-inflammatory signals reduce efferocytosis. For instance, both interferon (IFN)α, which is produced as a result of signaling via toll-like receptor (TLR)3, or direct TLR3 signaling using Poly:IC down regulates MerTK expression (as do LPS and IFNγ), but at the same time induces Axl expression in human macrophages, which may facilitate efferocytosis during viral infections (45). Signaling via the TLR-dependent upregulated TAM receptors may then interfere with TLR signaling via the induction of suppressor of cytokine signaling proteins (SOCS) 1 and 3, thus scaling down the proinflammatory response induced by the initial TLR signals (46).

## Relevance for Efferocytosis Mechanisms in Phagocytes in the Liver

Macrophage populations in adult tissues arise from different origins. Several tissue-resident macrophage populations, like Langerhans cells, microglia and Kupffer cells, are seeded in distinct waves during embryogenesis and are self-sustaining without input from the bone marrow during adulthood (47). The bone marrow also contributes to the myeloid/macrophage cell pool found in different tissues under homeostatic conditions and include Ly6C^+^ monocyte derived cells and dendritic cells. In particular in the liver, mas cytometry combined with gene expression analysis (48), shows that within the non-B, non-T CD45^+^ population the most abundant myeloid cells are dendritic cells, Kupffer cells and Ly6C^+^ monocytes (48). Distinct but small populations of plasmacytoid DC, neutrophils and basophils are also present. F4/80^+^MHCII^+^CD11b^+^ positive Kupffer cells comprise two populations additionally expressing CD11c or CD206, CD317 (plasmacytoid dendritic cell antigen-1, PCDA-1) and CD1d, pointing to a possible functional difference. Within Ly6C^+^ infiltrating monocytes, an F4/80 positive and F4/80 negative population are present. By intravital microscopy, the expression of F4/80 and/or CX_3_CR1, which is not expressed on Kupffer cells under homeostasis (49), shows that F4/80^+^ cells are exclusively to be found intravascularly (which may include both F4/80^+^ Kupffer cells and F4/80 expressing monocytes) and CX_3_CR1^+^ cells extravascularly in the parenchyma, suggesting that in addition to gene signatures another level of functionality is established via localization. Other markers that are used to distinguish tissue-resident Kupffer cells from recruited monocytes/neutrophils include the C-type lectin receptor Clec4f and the V-set and immunoglobulin domain-containing 4 (Vsig4) (50, 51). Interestingly, also the PS-receptor Tim4 is used for identification of liver resident Kupffer cells, indicating that these cells are set for efferocytosis under homeostatic conditions (50, 51). Thus, when studying the contribution of myeloid subsets to different liver diseases and/or injuries, the definition and identification of these different highly plastic myeloid subsets is challenging. Moreover, due to the large influx of pro-inflammatory myeloid subsets [neutrophils and inflammatory monocytes, but possibly also eosinophils (52)] the composition of the myeloid compartment at any given time during inflammatory liver disease can vary dynamically. We will focus on how local and recruited phagocytes are involved in the safeguarding of and return to immunological homeostasis in the Iiver and how their capacity for efferocytosis plays a role in the pathogenesis and resolution of liver diseases. Due to the highly divergent etiologies of liver disease, being either infectious, toxic or nutritional in origin, and depending on whether liver (immunological) function is being compromised acutely or in a chronic fashion, it is very well-possible that similar mechanisms controlling macrophage efferocytic function may have unexpected opposite effects.

First reports that intrahepatic phagocytes are capable of recognizing PS to trigger phagocytosis relate to the elimination of aged/damage erythrocytes from the circulation (4). The PS receptors Stabilin-1 and -2 expressed on LSEC are important for erythrocyte sequestration in this process (9). However, the removal of erythrocytes is blocked by clodronate-containing liposomes, indicating that Kupffer cells mediate actual phagocytosis. Also, aged erythrocytes that are taken up by Ly6C^+^ monocytes elsewhere in the circulation are carried to the liver as cargo to recover the iron from hemoglobin (53). Kupffer cells can express several receptors involved in PS recognition, such as MerTK (54) and Tim4 (53), or phagocytic receptors thought to assist in the uptake of PS exposed apoptotic cells like CD36 and scavenger receptors (55). The relevance for recognition of apoptotic cells and its downstream signaling events for potential treatment options in inflammatory diseases of the liver can be taken from experiments using apoptotic cells to treat inflammation and damage in D-galactosamine (D-GalN)/LPS induced liver damage (56). Here, adoptively transferred UV-irradiated splenocytes that are taken up by Kupffer cells prevent mortality in the D-GalN/LPS model, induces expression of IL-4, IL-10, and transforming growth factor beta (TGFβ) and suppresses expression of TNFα, IL-6, and IL-1β (56). Moreover, in a more controlled fashion liposomes expressing phosphatidyl serine may mimic apoptotic cell effects on phagocyte function in immune cells and hepatic disease (57, 58).

## Involvement of the Efferocytosis Machinery in Liver Disease

### Contribution of Efferocytosis Inducing Signals From Soluble Mediators Released by Apoptotic Cells

Early in the process of apoptosis, soluble mediators, such as ATP and UTP, act as damage-associated molecular patterns (DAMP) and “find-me” signals for efferocytes. In the liver, extracellular nucleotides trigger dichotomous responses; on the one hand, release of nucleotides by apoptotic cells after tissue injury initiates inflammation, and on the other hand, activation of non-parenchymal, liver-resident, and infiltrating efferocytes is a prerequisite for the activation of phagocytosis and the clearance of dead cells. Kupffer cells can detect and respond to the soluble mediator ATP released by apoptotic cells via expression of several P2Y receptors (59) and increase IL-6 production in response. Interestingly, however, Kupffer cells themselves also release ATP in response to LPS, which in an autocrine fashion promotes pro-inflammatory IL-6 release PY2_13_-dependently (59). Thus, attraction to apoptotic cells via ATP may lead to pro-inflammatory cytokine release by Kupffer cells. *In vivo* the recognition of and response to ATP in liver disease is more complex. After partial hepatectomy, both Kupffer cells and hepatocytes release ATP, which is important for the regenerative response in hepatocytes via direct signaling through the P2Y_2_ receptor (60), indicating that ATP release may have pro-inflammatory effects but at the same time may be indispensable for the initiation of hepatocyte proliferation necessary for liver regeneration. Depending on the longevity of ATP release and the expression of different purinergic receptors capable of recognizing ATP, the outcome of ATP release can be modulated. For instance, in the model of partial hepatectomy ATP release peaks within 15 min (59). In other models, where ATP is likely released over a prolonged time period, effects on intrahepatic phagocytes and disease progression may be different. In acetaminophen (APAP)-mediated liver injury, ATP (and also NAD) signaling via the purinergic P2X_7_ receptor mediates hepatotoxicity to a large extent as P2X_7_-deficient mice, and the use of selective P2X_7_ antagonists decreases APAP-dependent liver toxicity (61). Although direct ATP signaling induces proinflammatory signaling, such as IL-1β and IL-6 production by Kupffer cells (59, 61), ATP released during liver injury and inflammation *in vivo* can be further modulated to attenuate liver inflammation. The cell surface ectonucleotidase CD39 counteracts proinflammatory effects of ATP release by catalyzing the hydrolysis of ATP/ADP to AMP, which can then be further metabolized to adenosine that can results in A_2A_ receptor stimulation. In APAP liver injury, in both a caecal ligation and puncture (CLP) liver dysfunction model and a chemically induced biliary fibrosis model (by 3,5-diethoxycarbonyl-1,4-dihydrocollidine (DCC)-feeding), CD39-deficiency exacerbates disease (61–63). In DCC-dependent biliary fibrosis, CD39 deficiency in myeloid cells, via LysM-Cre-mediated excision of the CD39 gene, was sufficient for exacerbation of disease (63). As ATP signaling via the P2X_7_ receptor not only induces a proinflammatory phenotype in macrophages but also increases CD39 expression and CD39 activity in macrophages (62), this represents a mechanism to induce an anti-inflammatory pro-resolving response after initial pro-inflammatory signaling in macrophages during inflammation and injury in the liver.

The release of LPC from apoptotic cells and it signaling via the G-protein coupled receptor G2A (13) can function as a “find-me” signal for efferocytes. Additionally, LPC signaling via the G2A receptor may also enhance phagocytic activity by promoting phagosome maturation (64, 65) and induce an anti-inflammatory (M2-like) mRNA expression profile [arginase 1 (Arg1), chitinase-like 3 (Chil3), CD206] and cytokine/mediator production (IL-10, PGE_2_) (66). Preventing G2A signaling in CLP leads to higher mortality. However, inhibition Kupffer cell activity by gadolinium chloride treatment prevents this higher mortality in G2A^−/−^ mice (67), indicating that G2A signaling in Kupffer cells reduces mortality in this model. Unexpectedly, G2A deficiency leads to overproduction of not only pro-inflammatory IL-6 and TNFα but also anti-inflammatory IL-10 by Kupffer cells. In CLP, the increased production of IL-10 by G2A^−/−^ Kupffer cells is pivotal for the lack of bacterial clearance (and higher mortality) as this is reversed by either Kupffer cell depletion or IL-10 blockade (67).

The involvement of S1P as an efferocytic “find-me” signal regulating myeloid cells in liver disease has not been studied extensively. S1P is a major factor regulating immune cell migration to and from bone marrow and lymphoid organs and can signal through five S1PR. Reduced S1P serum levels in patients with liver cirrhosis and hepatocellular carcinoma (HCC) are associated with worse outcome and HBV co-infection (68, 69). However, in the experimental model of bile duct ligation, specific inhibition of S1PR_2/3_ diminishes liver injury and fibrosis due to the inhibition of monocyte migration from the bone marrow into the liver (70). Similarly, treatment with the S1P antagonist FTY in mice fed a NASH diet also prevents infiltration of inflammatory monocytes and reduces liver inflammation and injury (71). The disparity between low serum S1P in patients being a predictor for higher mortality and a decreased inflammatory liver injury due to inhibition of S1P signaling in experimental models, may be the result of different timing (prevention and onset in experimental settings vs. end-stage disease in patients) but may also have to with comorbidities in patients. For instance, hyperglycemia, associated with type II diabetes, directly affects intrahepatic myeloid cell function. In mice treated with the pancreatic β cell toxin streptozotocin, that induces hyperglycemia, Kupffer cells produce more IL-6 and TNFα and less IL-10 in response to APAP-mediated injury compared to normoglycaemic animals (72). In these hyperglycemic mice, an imbalance between AMPK and Akt signaling leads to enhanced reactive oxygen species (ROS) production promoting an M1 type instead of M2 type gene signature in Kupffer cells. Hyperglycemia also increases intrahepatic S1P and S1PR3 levels both in patients and experimental animals (73). Targeting ROS production with *N*-acetyl cysteine (72) or treatment with a S1PR3 antagonist (73) both reduces the proinflammatory polarization of Kupffer cells and is associated with less inflammatory liver damage, which suggests that the combination of these treatments might bring additional advantage to counteract pro-inflammatory skewing of intrahepatic macrophages due to hyperglycemia.

The CX_3_CR1 chemokine receptor is widely used to identify different populations of myeloid cells with the help of GFP-reporter mice (74). In the non-inflamed murine liver, CX_3_CR1 expression identifies a myeloid cell population residing outside of the hepatic sinusoids, whereas F4/80^+^ Kupffer cells are to be found intravascularly (48). Interestingly, single cell sequencing of healthy human hepatic immune cells did not reveal a similar division based on CX_3_CR1 expression, but rather identified two populations of CD68^+^ myeloid cells according to the expression of the macrophage receptor with collagenous structure (MARCO) receptor, with MARCO^pos^ cells transcriptionally resembling murine Kupffer cells and MARCO^neg^ having a rather inflammatory gene signature (75). It cannot, however, be excluded that CX_3_CR1 may play a role in human intrahepatic inflammatory responses, as human peripheral blood monocytes express high levels of CX_3_CR1 also in patients with liver disease (76). Although in the murine liver, CX_3_CR1 positive cells are present under homeostatic conditions, it is widely accepted that during inflammatory responses, the up-regulation of CX_3_CR1 on macrophages signifies their change from largely pro-inflammatory toward an anti-inflammatory, wound healing type of cell. Their anti-inflammatory properties could be enhanced by recognition of CX_3_CL1 that is released by apoptotic cells. The change of CCR2^high^CX_3_CR1^low^ inflammatory monocytes locally into CCR2^low^CX_3_CR1^high^ macrophages in a sterile liver injury model leads to optimal repair and is dependent on local production of IL-4 and IL-10 (77). However, in liver disease, also endothelial-related expression of CX_3_CL1 may attract CD16^+^ monocytes CX_3_CR1-dependently into areas with active inflammation (78) to dampen inflammation and promote resolution of injury. Fittingly, CX_3_CR1-deficient mice develop greater liver fibrosis in both chronic CCl_4_ and bile duct ligation models due to development of high amounts of TNFα- and NO-producing macrophages (79). Additionally, in the absence of CX_3_CR1 signaling, the pro-inflammatory circle is perpetuated, as expression of the anti-apoptotic B cell lymphoma associated oncogene 2 protein (Bcl-2) is not induced in CX_3_CR1-deficient macrophages.

### Relevance of Indirect Phosphatidyl Serine Recognizing Receptors for Efferocytosis in the Liver

Recognition of apoptotic cells is mediated by binding to phosphatidyl serine exposed on the outer leaflet cell membrane. PS receptors either engage directly with PS (e.g., Tim family, CD300 molecules, stabilins) or via bridging molecules that bind to PS (Gas6, Protein S and MGF-E8) that can then be recognized by certain integrins or receptors of the TAM family. Although Protein S is abundant in the circulation, Gas6 expression is more restricted. In acute CCl_4_-mediated liver injury, Gas6 expression is induced in liver macrophages, indicating a role for detection of apoptotic cells via Gas6 and TAM receptors in this model. However, Gas6-deficiency does not change acute liver damage after CCl_4_, but in the regenerative phase after CCl_4_ induced liver injury, hepatocyte proliferation is stunted, and a reduction in Kupffer cell numbers and infiltrating monocytes are observed, with concomitant reduction of pro-inflammatory cytokine production (80). Mechanistically, Gas6 can signal via Axl, which is also induced in the liver after acute CCl_4_ damage, and leads to autophagy induction, inhibition of NLRP3 inflammasome activation and subsequent reduction in IL-1β and IL-18 secretion, which is required to reinstall homeostasis (40).

The TAM (Tyro3-Axl-MerTK) family of receptor tyrosine kinases connects the recognition and removal of apoptotic cells to the induction of anti-inflammatory signaling in macrophages. General features of TAM-induced signaling include enhancement of phagocytosis, reduction of Type I IFN induced inflammation, inhibition of NLRP3 inflammasome activation via induction of autophagy and the promotion of anti-inflammatory cytokine production (34). The importance for these receptors in liver immune homeostasis becomes apparent in animals lacking all three TAM receptors. These mice develop an auto-immune hepatitis like disease, with high ALT, auto-antibodies and large immune cell infiltrates (81), which is dependent on bone-marrow derived cells. Individual TAM receptors can also modulate inflammatory response in the liver. In patients with acute liver failure MerTK-expressing macrophages expand, and migrate to the necrotic areas in the liver. Fittingly, in MerTK-deficient mice acute liver failure (ALF) due to APAP administration is aggravated and associated with a reduced number of hepatic macrophages and increased MPO^+^ neutrophils (54). This suggests that MerTK may be involved in the removal of apoptotic neutrophils in the course of ALF. By increasing the amounts of MerTK-expressing macrophages, through *in vivo* application of secretory leukocyte protease inhibitor (SLPI), the amount of MPO^+^TUNEL^+^ neutrophils after APAP administration are reduced and this may constitute a therapeutic approach in acute liver failure after accidental or deliberate paracetamol intoxication (54). However, in patients with decompensated cirrhosis and acute-on-chronic liver failure (ACLF) the opposite may hold true. In ACLF patients, high susceptibility to infections and accompanying monocyte dysfunction correlates with disease severity (82). Here, MerTK expressing monocytes and macrophages are highly increased and are associated with decreased TNFα and IL-6 production. Interestingly, the treatment of MerTK^+^ monocytes with a MerTK inhibitor increased proinflammatory cytokine production (82). Thus, either inhibiting or promoting expansion of MerTK expressing myeloid cells may be used to prevent immune paralysis in ACLF patients or enhance the clearance of necrotic material and apoptotic neutrophils in ALF patients, respectively.

Other non-TAM PS receptors may influence liver disease as well. The integrins αvβ3 and αvβ5, which recognize apoptotic cells via binding to the bridging molecule MFG-E8, can contribute to limiting fibrosis and the inhibition of proinflammatory mediator production in the chronic liver inflammation models of bile duct ligation and thioacetamine (TAA)-induced fibrosis (83). As the infiltration of macrophages with or without Cilengitide^®^ (an αvβ3 and αvβ5 inhibitor mimicking the RDG-peptide ligand for integrins) treatment remains the same in these models, it is not clear whether or not the effects of αvβ3 and αvβ5 inhibition targets efferocytosis mechanisms in macrophages.

### Relevance of Receptors Directly Recognizing Phosphatidyl Serine by Hepatic Macrophages for Liver Disease

The receptor stabilin-1 is well-known for mediating phosphatidyl serine-dependent removal of aged erythrocytes in the liver (9). In chronic CCl_4_ liver injury the absence of stabilin-1 aggravates fibrosis and delays resolution after termination of CCl_4_ administration. Furthermore, the phenotypic switch of pro-inflammatory macrophages to the pro-resolving Ly6C^low^ macrophage is impaired in stabilin-1-deficient mice (84). Interestingly, stabilin-1 expression in non-injured animals is restricted to endothelial cells, but in inflamed/injured livers an F4/80^+^stablilin-1^+^ macrophage population can be detected (84). In addition to stabilin-1 binding to PS to promote efferocytosis, fibrosis development can additionally be prevented via stabilin-1. Pro-fibrogenic chemokine production can be inhibited via binding of malondialdehyde (MDA)-LDL, generated from lipid peroxidation, to stabilin-1. Furthermore, during resolution of liver injury the absence of stabilin-1 prevents the change to Ly6C^low^ macrophages, which are thought to be pro-resolving macrophages (84). Besides stabilin-1, Tim4 can also bind directly to PS. Tim4 is over-expressed in hepatic macrophages of mice fed both a high fat diet (HFD) and methionine-choline deficient (MCD) diet (85) and its absence leads to increased inflammation and severe steatosis. Tim4 strongly binds to PS, but does not induce efferocytosis independently; it collaborates with TAM receptors to elicit efferocytosis (19, 86). Strikingly, signal transduction via the Tim4 cytoplasmic domain upon PS binding induces activation of AMPKα via LKB1, which is critical to initiate autophagy (85). What is more, the levels of Tim4 expression determine the degree of inhibition of NLRP3 protein complex expression. Tim4 deficiency, therefore, leads to high levels of IL-1β and IL-18 and proinflammatory damage in both NASH diets (85).

### Phagocytosis and Autophagy of Apoptotic Cells and Its Relevance for Liver Disease

The sensing and physical uptake of apoptotic cells by macrophages can enhance phagocytosis. The cargo that is taken up needs to be digested and processed appropriately and several intermediates that are generated during this process may have signaling functions to modulate macrophage function toward a pro-resolving phenotype. For instance, the TAM-receptors and also the Tim4 receptor connect the uptake of apoptotic cells to autophagy, which in its turn prevents inflammasome activation and ultimately the production of pro-inflammatory IL-1β and IL-18. This means that macrophages that are incapable of properly digesting their apoptotic cargo may display aberrant inflammatory functions or phagocytosis. For instance, macrophages that can engulf apoptotic cells but are defective in processing their apoptotic cargo, due to the lack of LC3 interacting glycoprotein non-metastatic melanoma protein B (Gpnmb) expression (87) are neither capable to increase pSTAT3-dependent IL-6 transcription and IL-10 release nor sustain efficient efferocytosis by increasing phagocytosis. Addition of exogenous IL-6 and IL-10 increased phagocytosis in Gpnmb-deficient macrophages, indicating that secondary cytokine production is an important mechanism induced by proper cargo digestion to regulate phagocyte function. Indeed, *in vivo* absence of Gpnmb reduces the amount of Ly6C^low^ restorative macrophages in acute APAP-mediated damage and also in chronic CCl_4_ induced fibrosis (87) which can be reversed by treatment with IL-6. Different secondary signaling events that influence macrophage efferocytosis and anti-inflammatory function may also depend on the appropriate digestion of apoptotic material. Chemical inhibition of the lysosomal acid lipase (LIPA) reduces cholesterol hydrolysis in macrophages, and leads to mitochondrial stress and pro-inflammatory NLRP3 and caspase-1 activation. Furthermore, anti-inflammatory LXR activation is reduced due to the limited production of oxysterols from cholesterol (88). *In vivo*, the inhibition of LIPA activity reduces efferocytosis of both stressed erythrocytes and apoptotic lymphocytes. Also, under conditions of hypercholesterolemia, in which efferocytosis is constrained (89), additional LIPA inhibition exacerbated this effect and this consequently leads to increased liver inflammation. Interestingly, LIPA inhibition does not only increases the production of *iNos* and *Il1b* mRNA, it is accompanied by an almost complete reduction of *Mertk, Axl*, and *Gas6* mRNA expression (88). The efferocytic uptake of apoptotic cells connects to autophagy, which is achieved via a process called LC3-complex associated phagocytosis (LAP) (38). This non-canonical autophagy pathway requires a so-called class III PI3K complex that comprises the core proteins Beclin1, UV radiation resistance-associated gene protein (UVRAG), and RUN domain containing, Beclin1-interacting protein (Rubicon) (90). During LAP, rubicon localizes to the single membrane LAPosome, which is decorated with LC3-II, and promotes PI(3)P production necessary to recruit downstream autophagic machinery, including the ubiquitin-conjugating enzymes autophagy related proteins ATG5, 7 and 12 (38, 90). As a results, alterations in LAP lead to stalled degradation of apoptotic cells, which is observed in autoimmune disorders such as systemic lupus erythematosus (SLE) (42). Furthermore, LAP that is active during efferocytosis instructs phagocytes to secrete the anti-inflammatory cytokines TGFβ and IL-10, and conversely to repress secretion of the pro-inflammatory cytokines IL-1β, IL-12, and TNFα (37, 91, 92).

In HFD livers, Kupffer cells show defective LC3-I and LC3-II induction after LPS treatment, which leads to overproduction of TNFα. This phenotypically overlaps with ATG7-deficiency in Kupffer cells, which leads to defective induction of autophagy and provokes increased TNFα release (93). Furthermore, ATG5-deficiency restricted to myeloid cells leads to higher inflammatory responses in liver injury models (CCl_4_, D-GalN/LPS) (94, 95). The lack of ATG5 expression results most prominently in NLRP3 and caspase-1 dependent IL-1β production, which promotes hepatic stellate cells to synthesize fibrotic factors and augments infiltration of Ly6C^high^ inflammatory monocytes and MPO^+^ neutrophils (94). Interestingly, the application of an IL-1R antagonist almost completely reverses injury and inflammatory phenotype due to ATG5 deficiency in mice, and offers a promising potential therapeutic approach in the prevention of liver fibrosis and limitation of acute toxic liver injury (94, 95). Thus, the way in which apoptotic cells are processed and digested in macrophages determines the signaling cascades that impose immunologically quiet anti-inflammatory responses on the liver and could be exploited to protect from liver inflammatory diseases.

### Interactions Between Phagocytes Can Affect Efferocytosis Efficiency in the Liver

In infections and inflammatory diseases, neutrophilic granulocytes are first responders and infiltrate into the inflamed injured tissue. Pro-inflammatory cytokine release, protease release and the formation of extracellular nuclear traps (NETs) during the process of NETosis are deployed by neutrophils to combat the infection/injury (96, 97). Neutrophils die partially after NETosis or spontaneously via apoptosis and are cleared by tissue-resident or infiltrating macrophages via efferocytosis and the ordered clearance of apoptotic neutrophils by hepatic macrophages is essential for resolution of liver injury. Expectedly, CCR2-antibody depletion of pro-inflammatory Ly6C^high^ monocytes during acute APAP injury increases the numbers of infiltrating neutrophils. However, these neutrophils are functionally changed and produce less ROS due to decreased NADPH2 expression and seem more long-lived as they upregulate pro survival factors (98). Reversely, changes in neutrophil activity may also influence efferocytic activity in macrophages. Mediators released by neutrophils during NETosis, may promote inflammatory signaling in macrophages but can also directly corrupt recognition of apoptotic cells by phagocytes: HMGB1, for example, can either bind to the PS-recognizing the RAGE receptor or directly binds to PS which interferes with recognition by other PS-binding proteins (26). Alcoholic liver disease (ALD) patients have elevated blood serum levels of HMGB1, which is secreted by neutrophils during NETosis. Interestingly, alcohol can directly increase NETosis by neutrophils (99) and thus in ALD patients the consumption of alcohol may directly affect pro- and anti-inflammatory pathways via HMGB1 in a dichotomous way: first, HMGB1 may act as a damage-associated molecular pattern protein (DAMP) that initiates pro-inflammatory TLR and NLRP3 signaling whilst simultaneously preventing efferocytosis via obscuring PS recognition. Furthermore, neutrophils can act as anti-inflammatory modulators that support the formation of pro-resolving macrophages via the release of micro-vesicles carrying microRNAs (100). MicroRNA-223, which inhibits NLRP3 inflammasome activation (101), is highly expressed by neutrophils. As such, miR-223-deficient mice are highly susceptible to liver inflammation in response to low-dose LPS injection or CCl_4_-mediated liver injury. During the resolution phase of inflammation after 1 week of CCl_4_ treatment or during the resolving phase after MCD diet feeding, depletion of Ly6G^+^ neutrophils impairs spontaneous resolution of liver injury and leads to the prevalence of pro-inflammatory Ly6C^high^ monocytes (100). Due to the lack of neutrophils that release miR-223 to be taken up by macrophages, NLPR3 inflammasome protein expression is exacerbated and liver inflammation persists, as pro-inflammatory macrophages cannot be converted into the CD163^pos^ anti-inflammatory, resolving type. Strikingly, this phenotype can be reversed by application of a miR-223 mimetic or the infusion of miR-223-compentent neutrophils. (100). In line with these observations, miR-223 is amongst the most prominent down regulated microRNAs in NASH patients (100, 102).

### Involvement of Efferocytosis Mechanisms in Liver Cancer

The tumor microenvironment (TME) is rich in immune cells of which macrophages and other myeloid cells are the most abundant (103, 104) and the presence of high amounts of CD68 and CD163 positive tumor-associated macrophages is associated with poor prognosis in a number of human cancers (105), including hepatocellular carcinoma (HCC) (106). The presence of tumor-associated macrophages (TAM) is also associated with the expression of efferocytosis receptors like MerTK, and the uptake of apoptotic tumor cells may promote further anti-inflammatory, tumor promoting polarization of TAMs (107). In hepatocellular carcinoma, molecules involved in efferocytosis are clinically relevant. The presence of soluble Axl in the serum of patients, for instance, is used as a biomarker for liver cirrhosis and HCC development (108, 109) and receptor tyrosine kinase inhibitors that can inhibit Axl function are currently being tested in therapeutic settings (110). However, here it is not clear whether Axl function in tumor-associated macrophages plays a role, as hepatocellular cancer cells themselves overexpress Axl, which provides survival advantages via activation of downstream signal transduction promoting epithelial to mesenchymal transition (EMT) (111). Similarly, overexpression of the bridging molecule Gas6 also promotes HCC metastasis via direct signaling through the Axl receptor in carcinoma cells (112). Contrary to this, several studies in preclinical cancer models have provided evidence that efferocytosis, mostly, but not exclusively, dependent on the TAM receptor MerTK is involved in promoting tumor progression, and several Axl, Tyro3, and MerTK targeting small molecular inhibitors are being tested in clinical trials (113). Tim3 expression on TAMs also correlates negatively with HCC patient survival, most likely via the promotion of pro-inflammatory IL-6 production by Tim3 expressing macrophages (114), which is thought to be essential for the development of hepatocellular carcinoma (115). Although the process of efferocytosis is associated with the development of an M2-like pro-resolving anti-inflammatory phenotype in tumor associated macrophages, which supports tumor progression, and oppositely the induction of pro-inflammatory M1-like macrophages promotes anti-tumor immunity (116), boosting the pro-inflammatory milieu in the absence of efferocytosis may contrarily promote tumorigenesis in the preneoplastic stage of carcinogenesis in the liver (117). Lastly, tumors may also evade immunosurveillance, by preventing recognition for efferocytosis. The “don't eat me” ligand CD47 is highly overexpressed in HCC samples and preventing interaction with SIRP1α by blocking anti-CD47 antibodies augments macrophage efferocytosis of HCC cells *in vitro* and diminishes HCC tumor growth in *in vivo* xenograft models (118, 119).

## Conclusion

The removal of dead cells poses a challenge to the maintenance of tolerance and the return to tissue integrity after inflammation or injury. Also in the liver, the maintenance of and return to immune homeostasis relies on functional efferocytosis (as summarized in Table 1), as demonstrated by the development of hepatic autoimmune disease in TAM receptor deficient mice. It has not been elucidated whether specific efferocytosis mechanisms exist in the liver. As during liver disease the most abundant cells involved in efferocytosis are infiltrating myeloid cells, general efferocytosis mechanisms known to be operative in macrophages have been investigated and found to play a role. However, under homeostatic conditions the most abundant myeloid cells in the liver are Kupffer cells. Due to their different ontogeny and expression of specific phagocytic receptors, such as the PS-receptor Tim4, complement receptor Vsig4 and the C-type lectin Clec4f, Kupffer cells may well-utilize additional pathways for efferocytosis under homeostasis. In liver disease, the use of global and cell specific knock-out mouse models for molecules involved in various aspects of efferocytosis has shown that efferocytosis mechanisms are heavily deployed in virtually all settings and stages of disease. The absence of functional efferocytosis in these genetic models can on the one hand lead to exacerbation of liver inflammation and injury or on the other hand to a lack and/or delay of resolution of inflammation and a insufficient return to tissue integrity. These opposite effects can be partly explained by the type of liver injury/inflammatory response (e.g., due to toxins or diet induced), but also by the length of the disease course and the timing of interference with efferocytosis. For instance, the inhibition of sufficient pro-inflammatory signaling can lead to defects in resolution, due to the fact that already early inflammatory responses set the stage for the induction of anti-inflammatory, pro-resolving pathways. The following inefficient induction of efferocytosis mechanisms impairs the clearance of damaged and apoptotic cells and subsequent loss of tissue homeostasis. In different etiologies of liver disease, it will be essential to understand the tempo-spatial contribution of pro- and anti-inflammatory and efferocytosis mechanisms in order to utilize these for treatment and prevention strategies in liver disease.

**Table 1:** Efferocytosis mechanisms in liver disease.

**Molecules directly or indirectly involved in efferocytosis**	**Cell-associated or soluble**	**Model of gastrointestinal disease, function in the liver, and/or function in efferocytosis**	**References**
**IMMUNE RECEPTORS**			
Vsig4	KC	Homeostasis	(51)
Tim4	KC	Homeostasis; in HFD and MCD, Tim4 deficiency: increase in inflammation and steatosis	(50, 53, 85)
Tim3	Liver mph	Hepatocellular carcinoma	(114)
**SCAVENGER RECEPTORS**			
Stabilin-1	LSEC, F4/80^+^ stabilin1^+^ mph	Removal of aged erythrocytes; CCl4; absence of Stabilin 1 aggravates fibrosis, delays in resolution	(9, 84)
Stabilin-2	LSEC	Removal of aged erythrocytes	(9)
CD36	KC, other phagocytes		(55)
**PURINERGIC G-PROTEIN COUPLED RECEPTORS RECEPTORS**			
P2Y receptors, P2Y13	KC	Expression in response to extracellular ATP, attraction of apoptotic cells, partial hepatectomy	(59)
P2Y2	HC	Partial hepatectomy, ATP release, and attraction of apoptotic cells	(60)
P2X7	KC	APAP, ATP release and attraction of apoptotic cells	(61)
**G-PROTEIN COUPLED RECEPTORS AND LIGANDS**			
G2A	KC	Enhancing phagosome maturation, CLP (cecal ligation and puncture)	(66, 67)
S1PR2/3		Bile duct ligation	(70)
S1P antagonist		NASH, S1P antagonist reduces monocyte infiltration, and reduces inflammation and injury	(71)
S1PR serum levels	Soluble, serum	Reduction of serum levels S1P, worse outcome in human liver cirrhosis, HCC, HBV	
S1P and S1PR3	Intrahepatic levels	Ischemia/reperfusion, hyperglycemia mice, and humans	(73)
S1PR3 antagonist		Reduction of inflammatory KC polarization	(73)
**CHEMOKINE RECEPTORS AND LIGANDS**			
Potenital: CX3CR1	KC, mph	recognition of CX3CL1 released by apoptotic cells, sterile liver injury	(77)
CX3CL1	LSEC	Potential attraction of CX3CR1 monocytes into areas of inflammation	(78)
CX3CR1		absence in deficient mice: greater liver fibrosis,CCl4 and BDL	(79)
**TAM FAMILY RECEPTORS AND LIGANDS**			
Gas6, Axl	Liver mph	CCl4, induction of Gas6/Axl expression, induction of autophagy, inhibition NLRP3 inflammasome	(80)
MertTK, Axl, Tyro3		MerTK^−/−^, Axl^−/−^ Tyro3^−/−^ mice, development of auto-immune hepatitis like disease, autoantibodies, immune cell infiltrates	(81)
MerTK	KC	MerTK deficient mice, ALF after APAP	(54)
MerTK		Human ACLF, decompensated cirrhosis, increase in MerTK expressing myeloid cells, immune paralysis, Hepatocellular carcinoma	(82, 107)
**INTEGRINS**			
avb3 and avb5		binding to MFG-E8, BDL, TAA-induced fibrosis	(83)
**INTRACELLULAR MOLECULES**			
Gpnmb	mph	Lack of Gpnmb impairs cytokine release and sustaining efferocytosis; in APAP, CCl4: reduction of restorative macrophages	(87)
Lysosomal acid lipase		Reduction Mertk, Axl, Gas6 expression, reduction of LXR activation, reduction of efferocytosis; in hypercholesteremia, increase in liver inflammation	(88, 89)
LC3-I and LC3-II deficiency	KC	HFD induces deficiency in LC3-I and LC3-II, overproduction of inflammatory cytokines	(93)
ATG7	KC	ATG7-deficiency in KC, increased release of inflammatory cytokines, defective autophagy	(93)
ATG5	Myeloid cells	Myeloid-restricted ATG5-deletion, hyperinfammation in CCl4/D-GalN/LPS, Hepatocellular carcinoma	(94, 95, 117)
HMGB1		PS or RAGE binding, impairs efferocytosis in ALD	(99)
miR-223	Neutrophils	CCl4, LPS-mediated liver injury: miR-223-releasing neutrophils promote generation of resolving macrophages; miR-223 downregulated in human NASH	(100, 102)
**OTHER**			
CD47	Non-phagocytes	Hepatocellular carcinoma	(118, 119)

## Author Contributions

AH, GT, and LD contributed to analysis of publications, drafting of the manuscript, and critical revision of the content.

### Conflict of Interest

The authors declare that the research was conducted in the absence of any commercial or financial relationships that could be construed as a potential conflict of interest.
